# Relationship between PD-L1 expression and prognostic factors in high-risk cutaneous squamous and basal cell carcinoma

**DOI:** 10.17305/bjbms.2022.7574

**Published:** 2022-06-30

**Authors:** Özden Yülek, Şebnem Batur, Kerem Özcan, Cansu Yol, Övgü Aydın Ülgen

**Affiliations:** 1 Department of Pathology, Siirt Research and Training Hospital, Siirt, Turkey; 2 Department of Pathology, Cerrahpasa Medical Faculty, Istanbul University-Cerrahpasa, Istanbul, Turkey; 3 Department of Pathology, Memorial Sloan Kettering Cancer Center, New York, United States; 4 Department of Pathology, Mehmet Akif Inan Training and Research Hospital, School of Medicine, University of Health Sciences, Sanliurfa, Turkey

**Keywords:** Cutaneous squamous cell carcinoma, basal cell carcinoma, PD-L1

## Abstract

This study aimed to investigate the programmed cell death-ligand 1 (PD-L1) expression in cutaneous squamous cell carcinoma (cSCC) and basal cell carcinoma (BCC) and its relationship with prognostic factors in tumors that are not in the head and neck region and are therefore relatively less exposed to the sun. This retrospective cross-sectional study included 25 invasive cSCC and 42 BCC cases with a diameter ≥2 cm located outside the head and neck region from 2010 to 2018. The biopsy samples were examined based on the membranous PD-L1 (22C3 clone) staining. Staining results were scored as follows: 0, no staining (negative); 1, <10% PD-L1 positivity of tumor cells; and 2, ≥10% PD-L1 positivity of tumor cells. PD-L1 positivity was not seen in any BCC cases, whereas 11 (44%) of cSCC cases were PD-L1 positive. No significant relationship was observed between PD-L1 expression and prognostic parameters, including tumor diameter, tumor depth, and lymphovascular or perineural invasion in the cSCC group. PD-L1 expression was not associated with prognostic factors in the early stages of BCC and SCC located outside the head and neck region. Therefore, investigating the PD-L1 expression seems to be more relevant in patients with advanced-stage disease.

## INTRODUCTION

Basal cell carcinoma (BCC) and cutaneous squamous cell carcinoma (cSCC) are the most common malignancies in humans [1]. Five-year survival rates of these tumors are considerably high, and classical treatment methods, such as surgery and radiotherapy, are generally sufficient. However, additional systemic treatment may be required for high-risk, locally-advanced, and metastatic BCC and cSCC [2-11]. Immunotherapy with anti-programmed cell death-ligand 1 (PD-L1) agents is a new treatment option for tumors that do not respond to classical treatment methods and is widely used for the treatment of malignant melanoma, lung carcinoma, renal cell carcinoma, and lymphoma [12-14].

PD-L1 and programmed cell death-1 (PD-1) activation play an important role in the tumors evasion of immune surveillance [15,16]. The best method to determine the PD-L1 status of a tumor is to detect PD-L1 expression by immunohistochemical analysis [15,17]. PD-L1 expression has been studied in different tumor types. However, data on PD-L1 expression in BCC and cSCC are limited.

Therefore, our study aimed to investigate the relationship between PD-L1 expression and prognostic parameters in patients diagnosed with cSCC and BCC that are not in the head and neck region, which are relatively less sun-exposed and supposed to have less mutation load [18,19].

## MATERIALS AND METHODS

**FIGURE 1:**
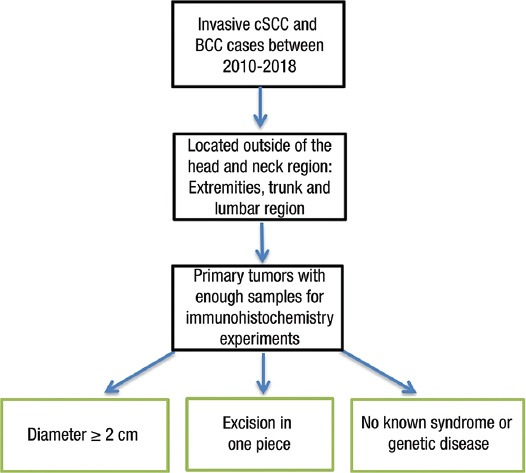
Patient selection algorithm for the study. cSCC: Cutaneous squamous cell carcinoma; BCC: Basal cell carcinoma.

Istanbul University-Cerrahpasa, Cerrahpasa Medical Faculty, Pathology Department, Database Archive was analyzed with computational software for cases that underwent excisional resection and were diagnosed with invasive cSCC and BCC from 1 January 2010 to 31 December 2018. The analysis found 467 cSCC and 1339 BCC cases. According to the 8^th^ edition of the American Joint Committee on Cancer Classification System, tumors over 2 cm in diameter have a higher potential to metastasize or recur locally [20]. Invasive primary tumors with a diameter of 2 cm or more that were excised in one piece and located outside the head and neck region were included in the study. The threshold of 2 cm was also applied to BCC for standardization, although there was no such system for BCC. Cases with different classification systems than cSCC, such as eyelid, vulva, and perianal regions, were excluded. Keratoacanthomas were also excluded from SCC cases based on the pre-2018 World Health Organization (WHO) classification [1]. Patients with syndromes that increase the likelihood of skin tumors, such as xeroderma pigmentosum and epidermolysis bullosa, were also excluded from the study. After evaluating the suitability of slides and blocks of these cases, 42 of 1339 BCC cases and 26 of 467 cSCC cases met the inclusion criteria and had available paraffin blocks. One cSCC case was excluded due to insufficient material. Finally, 25 cSCC and 42 BCC were found suitable for the study. The inclusion and exclusion criteria algorithms are depicted in Figure 1. Slides and paraffin blocks were obtained from the archive, and hematoxylin and eosin-stained slides were reevaluated, and diagnoses were reclassified according to the 2018 WHO Classification [1]. Tumor location, diameter, depth, perineural and vascular invasion, and metastasis status were documented from the software system. Representative sections with 4 μm thickness obtained from the paraffin-embedded blocks were stained with PD-L1 22C3 pharmDx assay (Dako, Carpinteria, CA) using a semi-automatic device (Dako Autostainer Link48 and Dako PT link). The obtained sections were deparaffinized and rehydrated. Then, the samples were incubated with anti-PD-L1 antibody (1:50 dilution; for 60 minutes incubation, 22C3 clone, and pharmDx antibody [Dako, Carpinteria, CA]).

**FIGURE 2:**
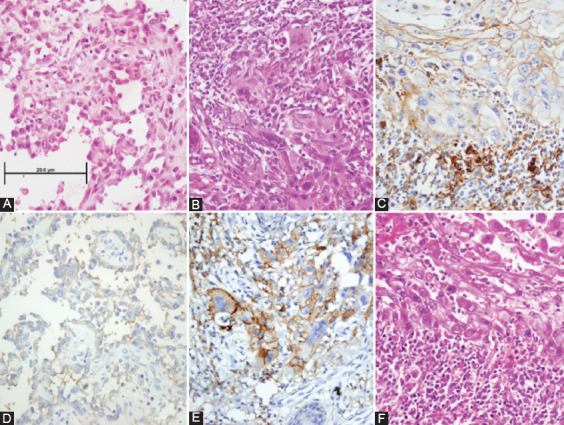
PD-L1 for cutaneous squamous cell carcinoma. (A), (B), (C) cSCC Hematoxylin & Eosin images, (D), (E), (F) PD-L1 staining examples of the cSCC cases, with same order as H&E (D) PD-L1 (+) cSCC (Score 1 [1%]), (E) PD-L1 (+) cSCC (Score 2 [10%]), and (F) PD-L1 (+) cSCC (Score 2 [20%]). All images were obtained at ×400 magnification (Scale bar = 20 μm). PD-L1: Programmed cell death-ligand 1; cSCC: Cutaneous squamous cell carcinoma.

Tonsillar crypt epithelial cell and follicular lymphoid cell staining were considered positive control. Although cytoplasmic PD-L1 staining was considered as positive in the earlier literature [21], the presence of membranous staining is accepted as positive in light of current literature [22,23]. Tumor cells with membranous staining were counted from at least 100 tumor cells and two different cutoff values were used - 1: <10% and 2: ≥10% (Figure 2).

### Ethical statement

The study was approved by the Istanbul University-Cerrahpasa Clinical Research Ethics Committee (Number: 31576681-605.99-73701, Date: 11 October 2018) and was conducted under the principles of the Helsinki Declaration.

### Statistical analysis

Statistical analysis was conducted using the Statistical Package for the Social Sciences (SPSS v 21.0) (Chicago, IL, USA). Descriptive statistics were expressed as mean, standard deviation, median, minimum, and maximum values for quantitative parameters, while percentages were calculated for non-quantitative parameters. The Kolmogorov–Smirnov and Shapiro–Wilk tests were used for data distribution. Non-parametric data were analyzed by the Mann–Whitney U-test and Chi-square tests. The Kruskal–Wallis test was utilized to compare non-parametric data with more than two variables followed by the Mann–Whitney U-test. The confidence interval was accepted as 95% and *p* < 0.05 was considered statistically significant.

## RESULTS

**FIGURE 3:**
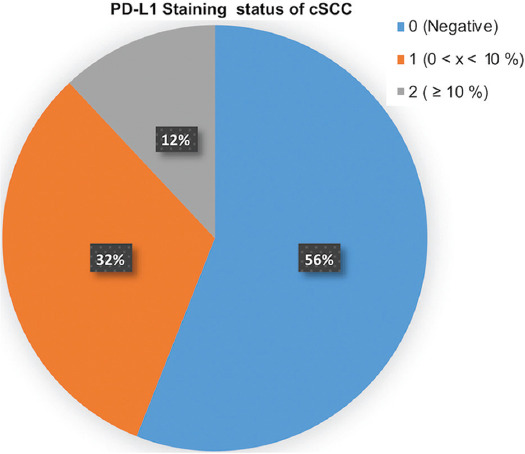
PD-L1 staining status of cSCC cases. PD-L1: Programmed cell death-ligand 1; cSCC: Cutaneous squamous cell carcinoma.

Of 25 cSCC cases, 11 (44%) were PD-L1 positive with varying degrees (Table 1). PD-L1 staining percentages are given in Figure 3. Of the cases showing PD-L1 expression, 7 (63.6%) were male and 4 (36.4%) were female. Statistical analyses revealed no significant difference between genders in terms of PD-L1 expression (*p* = 0.973).

**TABLE 1:**
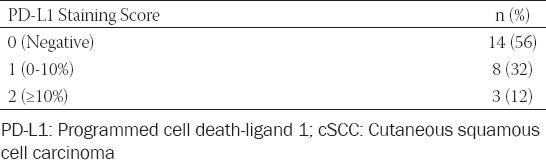
PD-L1 expression in cSCCs

**FIGURE 4:**
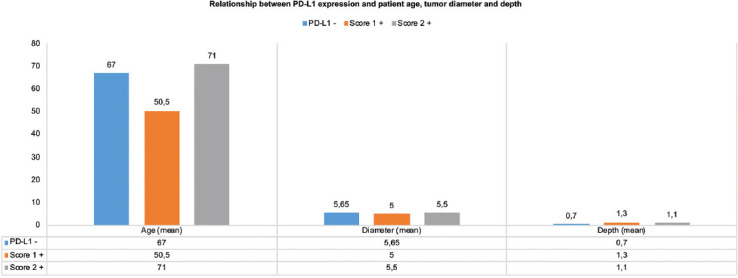
PD-L1 expression status of cSCC according to patient age, tumor diameter and depth. PD-L1: Programmed cell death-ligand 1; cSCC: Cutaneous squamous cell carcinoma.

Biopsies of 5 cases expressing PD-L1 were taken in 2017 and 2018. Analyzes of prognostic parameters, including tumor diameter, tumor differentiation grade, and depth of invasion, revealed a mean tumor diameter of 5.9 ± 3.3 cm in PD-L1 positive cases, which was slightly lower than in PD-L1 negative cases with a mean tumor diameter of 6.1 ± 4.1 cm (*p* = 0.851; Table 2). Meanwhile, a 10% cutoff level was applied, and staining scores were grouped as follows: Group 1 with a mean tumor diameter of 5.5 ± 2.3 cm and Group 2 with 7.1 ± 5.8 cm (*p* = 0.955; Table 2). Tumor invasion depth was 1.0 ± 0.5 cm in the PD-L1 positive group and 1.1 ± 1.0 cm in the PD-L1 negative group. PD-L1 status and its relationship between patients age, tumor diameter, and tumor depth are given Figure 4.

**TABLE 2:**
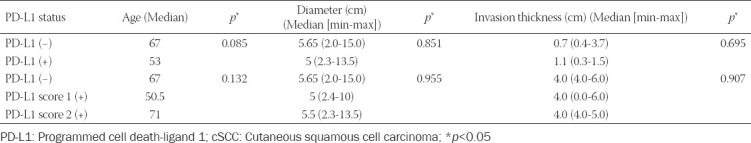
Relationship between PD-L1 expression status and age, tumor dimension, and invasion thickness parameters in cSCC

Six of 25 cases had lymphatic, blood vessel, or perineural invasion. Of the cases with lymph node metastases, two were PD-L1 negative, had at least average tumor size and Clark level V invasion thickness, and both had medium-grade differentiation levels.

The age of patients (*p* > 0.05; Table 2) and median tumor invasion thickness was similar in PD-L1 positive and PD-L1 negative group (*p* = 0.695; Table 2). The comparison within the PD-L1 positive group also revealed no significant differences in tumor invasion thickness (*p* = 0.907; Table 2).

There were variable amounts of inflammatory response around the tumor area in 22 of 25 cases. Groups based on inflammatory response revealed that 50% of cases had an intensive inflammatory response, 31.8% had moderate, and 18.2% had a mild inflammatory response. Among PD-L1 positive cases, 40% showed intensive, 40% showed moderate, and 20% showed mild inflammatory response. The statistical analyses revealed no significant relationship between the intensity of the inflammatory response and PD-L1 expression status (*p* = 0.508). Furthermore, no associations were observed between tumor differentiation degree with either PD-L1 positivity or PD-L1 negativity scores (Table 3).

**TABLE 3:**
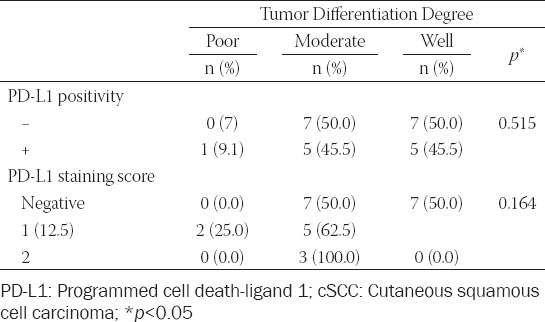
Relationship between tumor differentiation degree and PD-L1 positivity and PD-L1 expression pattern in cSCCs

None of the 42 cases in the BCC group was PD-L1 positive. Only one case, which had a peritumoral inflammatory infiltration showed diffuse PD-L1 staining in the inflammatory cells.

## DISCUSSION

BCC and cSCC are among the most common malignancies in humans. As previously discussed, the treatment of these tumors is generally not challenging. However, advanced treatment methods may be rarely required [24]. Lately, anti-PD-L1 immunotherapy is being used for treating various tumor types [17,25-29]. PD-L1 expression status is important for anti-PD-L1 therapies [24,25]. Meanwhile, limited studies and case reports on the use of immunotherapy in patients with BCC and cSCC mainly focus on tumors that are located in the head and neck region [10,30-36]. Our study aimed to investigate the PD-L1 status of BCC and cSCC located at places supposedly less exposed to sunlight compared with the head and neck.

A complementary immunohistochemical antibody remained for each therapeutic agent for anti-PD-L1 therapy in lung cancer. No evidence of complementary therapy is available for cutaneous non-melanocytic tumors. There are five Food and Drug Administration-approved PD-L1 antibodies in Blueprint studies. According to these studies, the sp142 clone shows less, and the 78-10 clone shows more PD-L1-positive tumor cells. There were no significant differences found in the other three clones [22,23]. Studies have compared immunohistochemical PD-L1 antibodies in malignant melanomas, but such studies for cSCC and BCC are unavailable [13].

Antibody clone divergency, biopsy sampling method, PD-L1 heterogeneity in the tumor, and different cutoff values in various studies make immunohistochemical analysis difficult [6,37-40]. The literature revealed an inverse correlation between the age of paraffin blocks for immunohistochemical PD-L1 study and PD-L1 expression. Blocks older than three years or even older than one year may show less PD-L1 antibody reactivity [41,42]. Five cases among our 11 PD-L1 positive cases were from 2017 to 2018, and the oldest case was from 2011.

Studies have reported the correlation between intensity of PD-L1 staining and the percentage of positive cells in malignant melanomas, although intensity of PD-L1 staining assessment is not accepted as a criterion for PD-L1 staining assessment [13,21]. Our study revealed only one cSCC case with more intense PD-L1 positivity than the other positive cases, which was also the case with the highest PD-L1 expression with a rate of 20%.

Studies on PD-L1 expression in non-melanocytic skin cancers are limited [43]. A novel study by Goto et al. compared PD-L1 expression of non-melanocytic skin tumors in 40 cases of the head and neck region and 40 cases outside the head and neck region and revealed a significant difference between PD-L1 positivity of these two groups using Roche sp263 clone. cSCC outside the head and neck region, which is supposed to be less exposed to the sun, had a positive PD-L1 positivity rate of 37.5%, whereas the sSCC in the head and neck region had a rate of 67.5% [19,44]. We studied cases located at the trunk, lumbar region, and extremities outside the head and neck region (less sun-exposed), but our positivity rates are lower than those of Goto et al. Our study did not compare the positivity rates of these two groups. Similar to the abovementioned study, Schaper et al. also reported higher PD-L1 positivity in cSCC of the head and neck region than in other areas [44].

PD-L1 expression in invasive cSCCs and their precursor lesions located in the head and neck region was found to be 26.9% in a study by Gambichler et al. [4]. PD-L1 expression percentage of the cSCC group in our series is compatible with these findings.

Primary cSCC cases located in the head and neck region represented 35% PD-L1 positivity in a study by Amoils et al. [45]. Our study revealed that all cases were primary tumors similar to their study and 44% of cases were detected as PD-L1 positive.

No significant relationships between PD-L1/PD-1 status and tumor differentiation degree in cSCCs were found by Oh et al., which is similar to our study [46]. However, they accepted cytoplasmic staining as positive in the well-differentiated cSCC group, while our study only accepted membranous staining as PD-L1 positivity. Slater and Googe revealed a positive relationship between the degree of PD-L1 expression and pathologic findings related to the risk of metastasis, such as large diameter, higher histologic grade, and tumor thickness [5]. Moreover, both lymph node metastatic cases in our study were compatible with this study, but there was no PD-L1 expression in these two cases in our study. The difference in these results may be because their cases had metastatic SCCs that were located in the head and neck region, where the sun exposure and mutation load were high [5].

There was a statistically significant relationship between survey and PD-L1 expression of >5% PD-L1 positivity in tumor-infiltrating lymphocites or tumor in a study using anti-PD-L1 sp263 clone in cSCC cases in the head and neck region [47]. Conversely, an increased metastasis risk with PD-L1 expression was reported in another study using sp142 clone [48]. Cutoff levels were 5% in the first study and 1% in the second. Varki et al. reported 26% PD-L1 positivity in 66 cSCC cases with sp142 clones using a 5% cutoff level. PD-L1 positivity was detected in 27% of immunocompetent cases and in 19% of immunosuppressed cases [49]. None of our cases had a known immunosuppression history, whereas 32% of our cases showed <10% and 12% showed >10% tumor cell PD-L1 positivity. Our results and results of Varki et al. were similar.

García-Pedrero et al. reported a correlation between increased lymph node metastasis risk and PD-L1 positivity using anti-PD-L1 E13LN clones in cSCC in the head and neck region. Concurrently, they observed increased PD-L1 expression in the tumor as the degree of differentiation decreased and the inflammatory response against the tumor increased, using 25% as a cutoff value to consider the expression positive [11].

PD-L1 positivity in 26.5% of cSCC cases with a cutoff value of ≥1% and 10.3% positivity with a cutoff value of ≥5% were reported by Schaper et al. Their study also indicated a positive correlation between the intensity of inflammation accompanying the tumor and the PD-L1 expression in both tumor cells and tumor-infiltrating lymphocites [44]. Our study revealed that 22 cSCC cases had various inflammatory response, which were grouped as mild, moderate, and severe as previously mentioned, but no relationship was found between PD-L1 expression and the density of peritumoral inflammation. Schaper et al. also reported no statistically significant relationship between PD-L1 expression and age, gender, tumor risk, and tumor differentiation grade, which is similar to our study. This may be due to the biological characteristics of the tumor. cSCC is relatively indolent than its counterparts in other organs, such as the lung and cervix. cSCC studies are limited and further studies with large series are needed for more information.

PD-L1 positivity was reported as 20% in low-risk cSCC and 70% in high-risk cSCC in a study by Slater and Googe. The relationship between PD-L1 expression profile and parameters indicating metastasis risks, such as tumor invasion thickness, tumor size, and histological grade, was analyzed and a statistically significant relationship was found in their study with 40 cSCC cases [5]. Our study revealed no statistically significant differences between parameters, which indicates a high-risk of metastasis and PD-L1 importance; however, large-scale studies with more cases are needed.

PD-L1 positivity in BCC with anti-PD-L1 2B11D11 clone was reported as 89.9% by Chang et al. [37]. This percentage rate is much higher than expected for any tumor. There is no information about immunohistochemical evaluation in their study. However, none of the BCC cases in our study were found to be PD-L1 positive. Gompertz-Mattar et al. revealed a 0.13% PD-L1 expression in the nodular type BCC in 156 BCC cases located mostly in the head and neck region. They did not use any cutoff level for evaluation. 22C3 clone was used similar to our study [35]. Furthermore, morpheaform and superficial types did not show any PD-L1 expression, which is also similar to our study. More studies with more cases are needed to understand the PD-L1 expression status in BCC with relevant and suitable antibodies.

Our study aimed to contribute to the literature by studying PD-L1 in BCC and cSCC. In selective cases, PD-L1 studies might be helpful, but in the early stages, PD-L1 status does not seem to be associated with prognostic factors, and it may be more valuable to investigate PD-L1 status in advanced-stage BCC and cSCC. Another pitfall is false negatives, which may occur as the age of the paraffin block gets older.

## CONCLUSIONS

The early stages of BCC and SCC PD-L1 expression does not seem to be associated with prognostic factors, and most likely PD-L1 positivity is more pronounced in advanced stages or metastases. Therefore, investigating PD-L1 expression in patients with advanced stages is more relevant.
